# Prenatal Exposure to Antiseizure Medications and Risk of Epilepsy in Children of Mothers With Epilepsy

**DOI:** 10.1001/jamanetworkopen.2023.56425

**Published:** 2024-02-26

**Authors:** Julie Werenberg Dreier, Jakob Christensen, Jannicke Igland, Mika Gissler, Maarit K. Leinonen, Håkon Magne Vegrim, Yuelian Sun, Torbjörn Tomson, Helga Zoega, Marte-Helene Bjørk, Rebecca L. Bromley

**Affiliations:** 1 The National Centre for Register-Based Research, Aarhus University, Aarhus V, Denmark; 2 Department of Clinical Medicine, University of Bergen, Bergen, Norway; 3 Department of Neurology, Aarhus University Hospital, Affiliated Member of the European Reference Network EpiCARE, Aarhus, Denmark; 4 Department of Clinical Medicine, Aarhus University, Aarhus, Denmark; 5 Department of Global Public Health and Primary Care, University of Bergen, Bergen, Norway; 6 Department of Health and Caring Sciences, Western Norway University of Applied Sciences, Bergen, Norway; 7 Knowledge Brokers, Finnish Institute for Health and Welfare, Helsinki, Finland; 8 Region Stockholm, Academic Primary Health Care Centre, Stockholm, Sweden; 9 Department of Molecular Medicine and Surgery, Karolinska Institute, Stockholm, Sweden; 10 Department of Clinical Epidemiology, Aarhus University Hospital, Aarhus, Denmark; 11 Department of Clinical Neuroscience, Karolinska Institutet, Stockholm, Sweden; 12 School of Population Health, Faculty of Medicine & Health, University of New South Wales Sydney, Sydney, Australia; 13 Centre of Public Health Sciences, Faculty of Medicine, University of Iceland, Reykjavík, Iceland; 14 Department of Neurology, Haukeland University Hospital, Bergen, Norway; 15 Division of Neuroscience, School of Biological Sciences, Faculty of Biology, Medicine and Health, The University of Manchester, Academic Health Science Centre, Manchester, United Kingdom; 16 Royal Manchester Children’s Hospital, Central Manchester University Hospitals NHS Foundation Trust, Manchester, United Kingdom

## Abstract

**Importance:**

Use of valproate and certain other antiseizure medications (ASMs) in pregnancy is associated with abnormal fetal brain development with potential long-term implications for the child.

**Objective:**

To examine whether use of valproate and other ASMs in pregnancy among mothers with epilepsy is associated with epilepsy risk in their children.

**Design, Setting, and Participants:**

This prospective, population-based register cohort study included singletons born to mothers with epilepsy in Denmark, Finland, Iceland, Norway, and Sweden from January 1, 1996, to December 31, 2017. Data analysis was performed from October 2022 to December 2023.

**Exposure:**

Redeemed prescription for an ASM from 30 days before pregnancy until birth.

**Main Outcomes and Measures:**

The main outcome was epilepsy in children, assessed using *International Statistical Classification of Diseases and Related Health Problems, Tenth Revision* diagnoses from hospital care. Adjusted hazard ratios (AHRs) and 95% CIs were estimated using Cox proportional hazards regression. Secondary analyses included dose-response analyses, analyses using children of mothers who discontinued ASM prior to pregnancy as the reference, and sibling analyses.

**Results:**

This cohort study included 38 663 children of mothers with epilepsy (19 854 [51.4%] boys). Children were followed up from birth; the mean length of follow-up was 7.2 years (range 0-22 years). Compared with 22 207 children of mothers not using an ASM in pregnancy, increased risks of epilepsy in children of mothers who used valproate in pregnancy (monotherapy: AHR, 2.18; 95% CI, 1.70-2.79; polytherapy: AHR, 2.10; 95% CI, 1.49-2.96) were observed. However, there was no dose-dependent association, and there was a similar risk of epilepsy in siblings who were exposed and unexposed to valproate (AHR, 0.95; 95% CI, 0.50-1.82). Prenatal exposure to topiramate monotherapy was associated with increased risk of epilepsy (AHR, 2.32; 95% CI, 1.30-4.16), and the risk was greater for higher doses, but the risk attenuated in comparisons with children of mothers who discontinued topiramate before pregnancy (AHR, 1.19; 95% CI, 0.26-5.44). Prenatal exposure to clonazepam monotherapy was also associated with increased epilepsy risk (AHR, 1.90; 95% CI, 1.16-3.12), but limited follow-up and low numbers precluded further analyses. No associations were observed for prenatal exposure to lamotrigine (AHR, 1.18; 95% CI, 0.95-1.47), levetiracetam (AHR, 1.28; 95% CI, 0.77-2.14), carbamazepine (AHR, 1.13; 95% CI, 0.85-1.50), or oxcarbazepine (AHR, 0.68; 95% CI, 0.44-1.05).

**Conclusions and Relevance:**

In this cohort study of children born to mothers with epilepsy, the associations found between prenatal exposure to certain ASMs and the child’s risk of epilepsy did not persist in sensitivity analyses, suggesting that maternal ASM use in pregnancy may not increase epilepsy risk in children beyond that associated with the maternal epilepsy itself. These findings are reassuring for women in need of treatment with ASM in pregnancy.

## Key Points

**Question:**

Is use of antiseizure medication in pregnancy among mothers with epilepsy associated with epilepsy risk in their children?

**Findings:**

In this cohort study of 38 663 children of mothers with epilepsy, there was a significant increase in the risk of epilepsy associated with prenatal exposure to valproate and certain other antiseizure medications, but these associations did not persist in sensitivity analyses (eg, in sibling analyses).

**Meaning:**

The finding that there is no association between prenatal exposure to valproate and other antiseizure medications and epilepsy risk in children of mothers with epilepsy in analyses more robust to confounding suggests that prenatal exposure to these medications does not add to the child’s risk of epilepsy beyond that associated with the maternal epilepsy itself.

## Teaser

This population-based cohort study assesses whether uses of antiseizure medications during pregnancy is associated with epilepsy risk in children.

## Introduction

Valproate is one of the most efficacious antiseizure medications (ASMs) especially for generalized epilepsy,^1^ but there is concern over the adverse-effect profile of the drug when used in pregnancy.^2^ In animal models, prenatal exposure to valproate was associated with increased risk of various morphological and functional alterations in fetal brain development^3,4,5,6,7,8,9^ and in human studies with poorer child neurodevelopmental outcomes such as developmental milestones,^10,11^ intellectual functioning,^11,12,13,14^ autism spectrum disorder (ASD),^13,14,15^ and language and memory functioning.^16,17,18^ Other ASMs may also impact the development and functioning of the brain, including phenobarbital and benzodiazepines such as diazepam and clonazepam,^9,19,20,21^ with neuropsychological effects having been described in human children.^22,23^ Preclinical animal data for other ASMs are fewer, but topiramate,^24,25^ carbamazepine,^26^ oxcarbazepine,^26^ and vigabatrin^9^ exposure in utero have all been associated with alterations in neuronal developmental processes.

Aberrations in the development of the fetal brain may increase risk of epilepsy.^27^ Given the alterations in fetal brain development experimentally induced by valproate and other ASMs in rodent models,^28^ it could be hypothesized that changes in neuron connectivity and excitability^5,8^ or from cytoarchitecture changes and/or migration abnormalities may lead to epileptogenic cortical areas.^6,26^ Previous work has suggested that children of mothers with epilepsy are at higher risk of developing epilepsy than are children of fathers with epilepsy,^29,30^ which has been coined the maternal effect.^29,30,31^ Examining whether use of ASMs among pregnant mothers with epilepsy may explain a maternal effect is nevertheless not straightforward in observational human studies. Genetic risk of epilepsy varies according to the type of maternal epilepsy, which informs the selection of a specific ASM. Valproate is likely to be used for generalized epilepsies,^1,32^ which have higher heritability than focal epilepsies, while sodium channel blockers, such as carbamazepine and oxcarbazepine, are used for focal epilepsies.^1,32^ With these considerations in mind, we aimed to examine whether use of valproate and other ASMs among pregnant mothers with epilepsy was associated with epilepsy in their children.

## Methods

### Study Design, Setting, and Population

This is a prospective, population-based cohort study carried out within the Nordic register-based study of antiepileptic drugs in pregnancy (SCAN-AED) project.^33^ The cohort consisted of pooled and harmonized data from nationwide health and social registers from Denmark, Finland, Iceland, Norway, and Sweden.^13,14^ Using the unique personal identification number assigned to all persons living in the country, we ensured accurate linkage of individual-level information across national registries. The relevant ethical and/or data-protection authorities in all participating countries approved the project. According to legislation in the Nordic countries, informed consent was waived because it is not required for register-based studies using pseudonymized data. We followed the Strengthening the Reporting of Observational Studies in Epidemiology (STROBE) reporting guideline.

In the present study, we included all live-born singletons of mothers with epilepsy from January 1, 1996, to December 31, 2017, in Denmark (from calendar years 1997 to 2017), Finland (from calendar years 1996 to 2016), Iceland (from calendar years 2004 to 2017), Norway (from calendar years 2005 to 2017), and Sweden (from calendar years 2006 to 2017) (eFigure in Supplement 1). Maternal epilepsy was defined by having a hospital contact with a recorded diagnosis of epilepsy (*International Statistical Classification of Diseases and Related Health Problems, Tenth Revision* [*ICD-10*] codes G40-G41 [all countries]) before delivery, having redeemed a prescription for an ASM with epilepsy given as the reason for reimbursement before delivery (Denmark since 2004, Norway, and Finland), or having any registered diagnosis of epilepsy in a medical birth register (all countries except Denmark).

### Prenatal ASM Exposure

Information on maternal use of ASMs was retrieved from the national prescription registers, which hold information on all redeemed prescriptions, including the date of dispensing, the Anatomical Therapeutic Chemical (ATC) classification code, and the number of dose units per pack. Children were considered prenatally exposed to an ASM if their mother had redeemed any prescription for medications with ATC codes N03A, N05BA09, or S01EC01 during the exposure window, which was defined as 30 days before the date of the last menstrual period (estimated using gestational age in days at birth) until birth. Monotherapy was defined as pregnancies in which the mothers had redeemed prescriptions for only 1 type of ASM within the exposure window and polytherapy as 1 or more prescriptions for 1 or more different ASMs during the exposure window. Polytherapy was divided into combinations with and without valproate. We calculated the mean daily dose of an ASM for each monotherapy as the sum of the defined daily doses from all prescriptions filled in the exposure window divided by the number of days in that period and created cutoffs at 50% and 100% of the defined daily dose (see the eMethods in Supplement 1). Since the duration of the ASM use might differ substantially, we also analyzed cumulative dose in the same period in a sensitivity analysis.

### Epilepsy in Children

We retrieved data on children with epilepsy from patient registers, which contained diagnostic information from inpatient admissions and outpatient visits in specialist care. We considered the children to be affected by epilepsy if they were registered with a diagnosis of epilepsy (*ICD-10* codes G40-G41). The date of epilepsy onset was defined as the admission date of the child’s first hospital contact with any registered diagnosis of epilepsy.

### Statistical Analysis

The children were followed up from birth until onset of epilepsy, emigration, death, or the end of follow-up on December 31, 2017, whichever came first. Data analysis was performed from October 2022 to December 2023. Using Cox proportional hazards regression, we estimated adjusted hazard ratios (AHRs) and corresponding 95% CIs for epilepsy, according to prenatal ASM exposure and ASM dose. In all models, we used the age of the child as the underlying time scale and allowed for separate baseline hazards for each country and birth year (as stratum) to account for country differences in diagnostic rates and calendar-period effects. In the basic adjusted model, a covariate was included for the sex of the child, whereas the fully adjusted models also included smoking in pregnancy, use of antidepressants (ATC code N06A) in pregnancy, and maternal characteristics assessed at the time of birth (age, parity, highest level of completed education, and psychiatric comorbidity). There was missing information on maternal smoking status (range, 6%-8%), maternal educational level (range, 2%-5%), and maternal parity (range, 0%-1%), and we included a separate missing category for these variables.^34^ Proportionality of hazards was assessed visually using log minus log plots. The threshold for statistical significance was set to 2-tailed .05, and there was no adjustment for multiple testing. We estimated the cumulative incidence of epilepsy using a nonparametric approach based on the subdistribution hazards, with death and emigration as competing events.

To better account for differences in maternal epilepsy type, we carried out a series of additional analyses. First, we restricted the population to children of mothers with active epilepsy (ie, those fulfilling epilepsy criteria from 1 year before pregnancy and until birth). Second, we performed a negative control exposure analysis using children whose mothers used an ASM in the year before (365 to 30 days before the last menstrual period) but not in pregnancy as the reference group (ie, assuming that the indication for treatment was similar in those who discontinued and continued a certain ASM). This analysis did not include data from Finland, as medication information was not available for the year prior to pregnancy. Third, we performed a sibling-controlled analysis based on siblings discordant for prenatal valproate exposure to better account for maternal characteristics, including epilepsy type and genetic characteristics. In these models, the maternal identification number was used as the stratum variable in the Cox proportional hazards regression analysis, and we included covariates for sex of the child and maternal characteristics that may have differed between pregnancies. Finally, we used ASD (*ICD-10* code F84 excluding F84.2-F84.4) and major congenital malformations^35^ as positive control outcomes for prenatal valproate exposure to evaluate the sensitivity of the algorithm used to estimate ASM dose and for the sibling design in detecting adverse effects. Log binomial regression models with similar adjustment variables were used to estimate adjusted relative risks (ARRs) of congenital malformations. Statistical analyses were performed using Stata, version 18 (StataCorp LLC).

## Results

We included 38 663 children of mothers with epilepsy (19 854 [51.4%] boys and 18 809 [48.7%] girls). Children were followed up from birth; the mean length of follow-up was 7.2 years (range, 0-22 years). Among these, 22 207 children (57.4%) were not prenatally exposed to an ASM, while 1952 (5.0%) were exposed to valproate in monotherapy and 822 (2.1%) to valproate in polytherapy. Characteristics of children with prenatal exposure to valproate are provided in Table 1 and to other ASMs are provided eTable 1 in Supplement 1.

**Table 1.** Characteristics of Children of Mothers With Epilepsy by Prenatal Valproate Exposure

Characteristic	Children of mothers with epilepsy, No. (%)		
	Without ASM (n = 22 207)	With valproate monotherapy (n = 1952)	With valproate polytherapy (n = 822)
Country of birth			
Denmark	9385 (42.3)	418 (21.4)	179 (21.7)
Finland	1225 (5.5)	976 (50.0)	338 (41.1)
Iceland	91 (0.4)	13 (0.7)	10 (1.2)
Norway	6101 (27.5)	190 (9.7)	106 (12.9)
Sweden	5405 (24.3)	355 (18.2)	190 (23.1)
Year of birth			
1996-1999	401 (1.8)	227 (11.6)	90 (10.9)
2000-2004	1457 (6.6)	392 (20.1)	150 (18.2)
2005-2009	6482 (29.2)	678 (34.7)	233 (28.3)
2010-2014	8552 (38.5)	510 (26.1)	230 (27.9)
2015-2017	5315 (23.9)	145 (7.4)	120 (14.6)
Sex of child			
Female	10 821 (48.7)	963 (49.3)	402 (48.9)
Male	11 386 (51.3)	989 (50.7)	420 (51.1)
Gestational age at birth (<37 wk), wk	1501 (6.8)	121 (6.2)	61 (7.4)
Birth weight (<2500 g), g	1001 (4.5)	91 (4.7)	54 (6.6)
Maternal age, y			
<20	498 (2.2)	82 (4.2)	19 (2.3)
20-24	3608 (16.2)	345 (17.7)	136 (16.5)
25-29	6945 (31.3)	576 (29.5)	261 (31.7)
30-34	6960 (31.3)	619 (31.7)	239 (29.0)
35-39	3483 (15.7)	280 (14.3)	138 (16.8)
≥40	713 (3.2)	50 (2.6)	30 (3.6)
Maternal parity			
0	9834 (44.3)	878 (45.0)	390 (47.4)
1	7713 (34.7)	663 (34.0)	262 (31.8)
≥2	4607 (20.7)	390 (20.0)	164 (19.9)
Missing	53 (0.2)	21 (1.1)	7 (0.9)
Maternal educational level			
Compulsory	5832 (26.3)	398 (20.4)	239 (29.0)
Secondary or preuniversity	9508 (42.8)	1029 (52.7)	421 (51.2)
Bachelor’s degree	4230 (19.0)	287 (14.7)	99 (12.0)
Master’s degree or PhD	2057 (9.3)	138 (7.1)	39 (4.7)
Missing	580 (2.6)	100 (5.1)	25 (3.0)
Smoked in pregnancy			
No	16 824 (75.8)	1388 (71.1)	591 (71.8)
Yes	3931 (17.7)	415 (21.3)	164 (19.9)
Missing	1452 (6.5)	149 (7.6)	68 (8.3)
Used antidepressants in pregnancy	1631 (7.3)	101 (5.2)	48 (5.8)
Maternal psychiatric disorder^a^	5974 (26.9)	229 (11.7)	153 (18.6)

Abbreviation: ASM, antiseizure medication.

^a^ Any maternal diagnosis of a psychiatric disorder (*International Statistical Classification of Diseases and Related Health Problems, Tenth Revision* codes F00-F99) registered in the patient or hospital registers before birth of the child, which included inpatient contacts since 1994 and outpatient or emergency department contacts since 1995 (Denmark); inpatient contacts since 1996 and outpatient contacts in public hospitals since 1998 (Finland); inpatient contacts since 2002 and outpatient contacts since 2010 (Iceland); outpatient and inpatient contacts and data from contracted private specialists since 2008 (Norway); and outpatient and inpatient contacts since 2005 (Sweden).

The cumulative risk of epilepsy at 15 years of age varied significantly according to prenatal ASM exposure (Table 2), from 3.2% (95% CI, 2.8%-3.6%) in those with no prenatal ASM exposure to 9.1% (95% CI, 7.4%-10.9%) in those with prenatal exposure to valproate monotherapy and 8.5% (95% CI, 6.1%-11.5%) in those exposed to valproate polytherapy. In fully adjusted models with children of mothers with epilepsy who did not use an ASM in pregnancy as the reference, we observed significantly increased risks of epilepsy in children with prenatal exposure to valproate (monotherapy: AHR, 2.18; 95% CI, 1.70-2.79 and polytherapy: AHR, 2.10; 95% CI, 1.49-2.96), topiramate monotherapy (AHR, 2.32; 95% CI, 1.30-4.16), clonazepam monotherapy (AHR, 1.90; 95% CI,1.16-3.12), and polytherapy without valproate (AHR, 1.39; 95% CI, 1.04-1.84) (Table 2). No increased risks (at a significance level of 0.05) were observed in children with prenatal monotherapy exposure to lamotrigine (AHR, 1.18; 95% CI, 0.95-1.47), levetiracetam (AHR, 1.28; 95% CI, 0.77-2.14), carbamazepine (AHR, 1.13; 95% CI, 0.85-1.50), or oxcarbazepine (AHR, 0.68; 95% CI, 0.44-1.05). When restricting the sample to 25 138 children of mothers with active epilepsy, associations between valproate and topiramate and risks of epilepsy in children with prenatal exposure remained (eTable 2 in Supplement 1).

**Table 2.** Association of Prenatal Exposure to Valproate and Other ASMs With Childhood Epilepsy^a^

ASM exposure	Children of mothers with epilepsy		Incidence rate per 10 000 person-years (95% CI)	AHR (95% CI)		Cumulative incidence, % (95% CI)	
	Total No. (N = 38 663)	With epilepsy, No.					
				Basic adjusted^b^	Fully adjusted^c^	Age 10 y	Age 15 y
No ASM	22 207	390	26.5 (24.0-29.2)	1 [Reference]	1 [Reference]	2.5 (2.2-2.8)	3.2 (2.8-3.6)
Any ASM	16 456	497	37.7 (34.5-41.2)	1.34 (1.16-1.54)	1.38 (1.19-1.59)	3.6 (3.3-4.0)	5.4 (4.8-6.0)
Monotherapies^d^							
Valproate	1952	122	62.8 (52.6-75.0)	2.15 (1.68-2.76)	2.18 (1.70-2.79)	5.9 (4.8-7.2)	9.1 (7.4-10.9)
Lamotrigine	5289	104	30.4 (25.1-36.8)	1.15 (0.93-1.43)	1.18 (0.95-1.47)	3.1 (2.4-3.8)	NA
Levetiracetam	1061	16	34.9 (21.4-57.0)	1.27 (0.76-2.11)	1.28 (0.77-2.14)	NA	NA
Carbamazepine	2664	81	30.4 (24.4-37.8)	1.05 (0.79-1.39)	1.13 (0.85-1.50)	2.9 (2.2-3.7)	4.5 (3.4-5.6)
Oxcarbazepine	1460	27	18.6 (12.7-27.1)	0.65 (0.42-1.02)	0.68 (0.44-1.05)	1.6 (1.0-2.5)	2.7 (1.7-4.1)
Topiramate	290	12	62.0 (35.2-109.1)	2.37 (1.33-4.23)	2.32 (1.30-4.16)	NA	NA
Clonazepam	339	18	49.3 (31.1-78.3)	1.89 (1.16-3.08)	1.90 (1.16-3.12)	3.8 (2.0-6.4)	7.1 (3.8-11.7)
Polytherapies^d^							
Without valproate	2090	62	40.0 (31.2-51.3)	1.42 (1.07-1.89)	1.39 (1.04-1.84)	4.4 (3.4-5.7)	NA
With valproate	823	47	63.0 (47.3-83.9)	2.14 (1.52-3.00)	2.10 (1.49-2.96)	5.7 (4.1-7.8)	8.5 (6.1-11.5)

Abbreviations: AHR, adjusted hazard ratio; ASM, antiseizure medication; NA, not analyzed due to low numbers or insufficient follow-up time.

^a^ Based on 5 Nordic countries (Denmark, Finland, Iceland, Norway, and Sweden) from 1996 to 2017.

^b^ Adjusted for year of birth, sex of the child, and country of birth.

^c^ Additionally adjusted for maternal age, parity, educational level, smoking in pregnancy, use of antidepressants in pregnancy, and maternal psychiatric comorbidity.

^d^ Numbers do not sum to any ASM exposure because the following monotherapies are not included due to low numbers: gabapentin, pregabalin, eslicarbazepine, lacosamide, acetazolamide, phenobarbital, and phenytoin.

Epilepsy risk associated with prenatal valproate exposure was not dose dependent (Table 3): compared with children of mothers with no ASM use in pregnancy, risk was similar for children prenatally exposed to low (<750 mg/d: AHR, 2.18; 95% CI, 1.58-3.02), medium (750-1499 mg/d: AHR, 2.19; 95% CI, 1.58-3.02), or high (≥1500 mg/d: AHR, 2.14; 95% CI, 1.30-3.50) doses of valproate. Analyses of topiramate and clonazepam were limited by the lower number of exposed children, but for these ASMs, epilepsy risk was highest in children prenatally exposed to higher doses (topiramate ≥150 mg/d: AHR, 4.88; 95% CI, 2.47-9.62 and clonazepam ≥4 mg/d: AHR, 3.66; 95% CI, 1.48-9.05). For the remaining monotherapies, we did not observe a pattern of dose dependency. Analyses considering the cumulative dose of prenatal ASM exposure showed comparable findings (eTable 3 in Supplement 1). Furthermore, in sensitivity analyses, prenatal valproate exposure was associated with a clear dose-dependent risk pattern for ASD (low: AHR, 1.72; 95% CI, 1.12-2.65; medium: AHR, 2.92; 95% CI, 2.04-4.17; and high: AHR, 3.63; 95% CI, 2.20-5.98) and for major malformations (low: ARR, 1.30; 95% CI, 0.98-1.72; medium: ARR, 1.80; 95% CI, 1.41-2.30; and high: ARR, 5.23; 95% CI, 4.19-6.52).

**Table 3.** Association of Different Doses of Prenatal Exposure to Valproate and Other ASMs With Childhood Epilepsy^a^

ASM monotherapy exposure, mg/d	Children of mothers with epilepsy		Incidence rate, per 10 000 person-years (95% CI)	AHR (95% CI)		Cumulative incidence, age 10 y, % (95% CI)
	Total No. (N = 38 663)	With epilepsy, No.				
				Basic adjusted^b^	Fully adjusted^c^	
No ASM	22 207	390	26.5 (24.0-29.2)	1 [Reference]	1 [Reference]	2.5 (2.2-2.8)
Valproate						
<750	774	48	63.5 (47.8-84.2)	2.24 (1.62-3.09)	2.18 (1.58-3.02)	6.0 (4.3-8.1)
750-1499	888	55	62.4 (47.9-81.2)	2.11 (1.53-2.92)	2.19 (1.58-3.02)	5.6 (4.1-7.5)
≥1500	290	19	62.4 (39.8-97.9)	2.04 (1.24-3.33)	2.14 (1.30-3.50)	6.7 (4.0-10.2)
Lamotrigine						
<150	1480	29	26.7 (18.6-38.4)	1.04 (0.71-1.52)	1.01 (0.69-1.48)	2.9 (1.9-4.2)
150-299	1617	38	35.7 (26.0-49.0)	1.36 (0.97-1.91)	1.44 (1.03-2.03)	3.3 (2.3-4.7)
≥300	2192	37	29.1 (21.1-40.2)	1.07 (0.76-1.51)	1.12 (0.79-1.58)	3.0 (2.0-4.3)
Levetiracetam						
<750	225	5	54.1 (22.5-129.9)	1.93 (0.79-4.73)	1.86 (0.75-4.57)	NA
750-1499	330	5	36.8 (15.3-88.3)	1.38 (0.57-3.37)	1.38 (0.56-3.36)	NA
≥1500	506	6	26.1 (11.7-58.1)	0.93 (0.41-2.10)	0.98 (0.43-2.21)	NA
Carbamazepine						
<500	800	27	35.0 (24.0-51.0)	1.18 (0.78-1.79)	1.21 (0.79-1.83)	2.7 (1.6-4.1)
500-999	1270	34	25.2 (18.0-35.3)	0.85 (0.58-1.26)	0.95 (0.64-1.40)	2.7 (1.9-3.9)
≥1000	594	20	36.5 (23.6-56.6)	1.29 (0.81-2.05)	1.42 (0.89-2.28)	3.7 (2.1-6.1)
Oxcarbazepine						
<500	232	<5^d^	NA	NA	NA	NA
≥500	1228	20^d^	19.8 (13.3-29.6)	0.70 (0.44-1.11)	0.73 (0.46-1.17)	1.6 (0.9-2.6)
Topiramate						
<150	188	<5^d^	NA	NA	NA	NA
≥150	102	10^d^	139.7 (72.7-268.5)	4.79 (2.44-9.39)	4.88 (2.47-9.62)	NA
Clonazepam						
<4	292	13	41.5 (24.1-71.4)	1.61 (0.92-2.84)	1.61 (0.91-2.85)	3.0 (1.4-5.7)
≥4	47	5	97.2 (40.4-233.4)	3.43 (1.39-8.49)	3.66 (1.48-9.05)	NA

Abbreviations: AHR, adjusted hazard ratio; ASM, antiseizure medication; NA, not analyzed due to low numbers or insufficient follow-up time.

^a^ Based on 5 Nordic countries (Denmark, Finland, Iceland, Norway, and Sweden) from 1996 to 2017.

^b^ Adjusted for year of birth, sex of the child, and country of birth.

^c^ Additionally adjusted for maternal age, parity, educational level, smoking in pregnancy, use of antidepressants in pregnancy, and maternal psychiatric comorbidity.

^d^ Rounded for data privacy reasons.

Analyses using children of mothers who discontinued ASM treatment before pregnancy as the reference are shown in Table 4. In these analyses, the AHR was 1.69 (95% CI, 0.91-3.16) for risk of epilepsy in children of mothers who used valproate in pregnancy compared with children of mothers who discontinued valproate before pregnancy. There was no difference in epilepsy risk between children of mothers who used topiramate in pregnancy compared with children of mothers who discontinued topiramate before pregnancy (AHR, 1.19; 95% CI, 0.26-5.44). For most other ASMs, data were too limited for analyses.

**Table 4.** Association of Prenatal Exposure to Valproate and Other ASMs With Childhood Epilepsy Among Children Whose Mothers Discontinued ASMs in the Year Before Pregnancy^a^

ASM monotherapy exposure	Total children, No. (N = 32 780)	With epilepsy, No.	Incidence rate, per 10 000 person-years (95% CI)	AHR (95% CI)		Cumulative incidence, age 10 y, % (95% CI)
				Basic adjusted^b^	Fully adjusted^c^	
Valproate						
Discontinued in the year before pregnancy	324	13	43.4 (25.2-74.8)	1 [Reference]	1 [Reference]	4.6 (2.5-7.7)
Use in pregnancy	976	62	65.9 (51.4-84.6)	1.56 (0.84-2.90)	1.69 (0.91-3.16)	6.4 (4.8-8.2)
Lamotrigine						
Discontinued in the year before pregnancy	914	27	43.0 (29.5-62.7)	1 [Reference]	1 [Reference]	4.0 (2.5-6.0)
Use in pregnancy	4766	85	27.8 (22.5-34.4)	0.63 (0.40-0.98)	0.65 (0.42-1.03)	2.8 (2.2-3.5)
Levetiracetam						
Discontinued in the year before pregnancy	110	<5	NA	1 [Reference]	1 [Reference]	NA
Use in pregnancy	886	16	42.0 (25.7-68.5)	NA	NA	NA
Carbamazepine						
Discontinued in the year before pregnancy	255	<5	NA	1 [Reference]	1 [Reference]	NA
Use in pregnancy	1618	39	28.9 (21.1-39.5)	NA	NA	3.1 (2.2-4.3)
Oxcarbazepine						
Discontinued in the year before pregnancy	107	<5	NA	1 [Reference]	1 [Reference]	NA
Use in pregnancy	512	16	28.3 (17.3-46.2)	NA	NA	2.9 (1.6-4.9)
Topiramate						
Discontinued in the year before pregnancy	226	5	35.9 (15.0-86.3)	1 [Reference]	1 [Reference]	NA
Use in pregnancy	251	9	53.2 (27.7-102.3)	1.52 (0.47-4.94)	1.19 (0.26-5.44)	NA
Clonazepam						
Discontinued in the year before pregnancy	123	<5	NA	1 [Reference]	1 [Reference]	NA
Use in pregnancy	324	16	45.8 (28.1-74.8)	NA	NA	3.3 (1.6-6.0)

Abbreviations: AHR, adjusted hazard ratio; ASM, antiseizure medication; NA, not analyzed due to low numbers or insufficient follow-up time.

^a^ Based on 4 Nordic countries (Denmark, Iceland, Norway, and Sweden; medication information was not available from Finland for the year prior to pregnancy) from 1996 to 2017.

^b^ Adjusted for year of birth, sex of the child, and country of birth.

^c^ Additionally adjusted for maternal age, parity, educational level, smoking in pregnancy, use of antidepressants in pregnancy, and maternal psychiatric comorbidity.

Among 25 608 mothers with epilepsy included in this study, we identified 258 (1.0%) who used valproate in at least 1 pregnancy and no ASM in at least 1 other pregnancy. In these sibling sets, we observed no difference in epilepsy risk between children with prenatal valproate exposure and their unexposed sibling (AHR, 0.58; 95% CI, 0.23-1.46) (Table 5). When considering 418 sibling sets in which the mother used valproate in at least 1 pregnancy and no valproate in at least 1 other pregnancy (ie, including mothers using other ASMs than valproate, or no ASMs), we still observed no difference (AHR, 0.95; 95% CI, 0.50-1.82). In sensitivity analyses based on the 418 sibling sets of mothers using valproate in 1 pregnancy and no valproate in another pregnancy, we found a higher risk of ASD (eTable 4 in Supplement 1) and major malformations (eTable 5 in Supplement 1) in the sibling exposed to valproate vs the unexposed sibling (ASD: AHR, 6.41 [95% CI, 2.00-20.58] and major malformations: 6.1% of unexposed siblings vs 9.3% of exposed siblings; ARR, 1.66 [95% CI, 1.03-2.67]). Due to limited numbers, we were unable to undertake sibling analyses for topiramate and clonazepam.

**Table 5.** Sibling Analyses of the Association of Prenatal Valproate Exposure With Childhood Epilepsy^a^

ASM exposure	Sibling sets of mothers with epilepsy		Incidence rate, per 10 000 person-years (95% CI)	AHR (95% CI)	
	Total No. (N = 13 886)	With epilepsy, No.		Unadjusted	Adjusted^b^
Sibling sets of mothers using valproate in at least 1 pregnancy and no ASM in at least 1 other	258	NA	NA	NA	NA
Pregnancies with no ASM use	374	16	44.0 (27.0-71.9)	1 [Reference]	1 [Reference]
Pregnancies with valproate use	312	16	47.3 (29.0-77.3)	0.59 (0.24-1.42)	0.58 (0.23-1.46)
Sibling sets of mothers using valproate in at least 1 pregnancy and no valproate in at least 1 other	418	NA	NA	NA	NA
Pregnancies with no valproate use	604	23	40.9 (27.2-61.6)	1 [Reference]	1 [Reference]
Pregnancies with valproate use	529	31	54.2 (38.1-77.1)	1.08 (0.57-2.04)	0.95 (0.50-1.82)

Abbreviations: AHR, adjusted hazard ratio; ASM, antiseizure medication; NA, not applicable.

^a^ Based on 5 Nordic countries (Denmark, Finland, Iceland, Norway, and Sweden) from 1996 to 2017.

^b^ Adjusted for sex of the child, maternal age, parity, smoking in pregnancy, and use of antidepressants in pregnancy.

## Discussion

This is the first study, to our knowledge, examining whether prenatal exposure to valproate and other ASMs may be associated with epilepsy risk in children of mothers with epilepsy. In this cohort study of singletons in Denmark, Finland, Iceland, Norway, and Sweden, we found that children of mothers with epilepsy who used valproate, topiramate, or clonazepam in pregnancy were more likely to develop epilepsy compared with children of mothers with epilepsy not using any ASM in pregnancy. However, sensitivity analyses in siblings and children of mothers who discontinued ASM treatment prior to pregnancy suggest that the associations with valproate and topiramate use found in the initial analyses were most likely explained by differences in some underlying factors, such as the heritability of the maternal epilepsy. For clonazepam, the analyses were limited by low numbers, and it remains unclear to which extent the association with epilepsy could be explained by similar factors or by the medication itself.

In our cohort, nearly 1 in 10 children of mothers with epilepsy using valproate in pregnancy had developed epilepsy by the age of 15 years. However, risk of epilepsy did not differ according to whether the mother had used high, medium, or low doses of valproate, and risk of epilepsy was similar in siblings exposed and unexposed to valproate. These analyses suggest that the increased risk of epilepsy found in children with prenatal valproate exposure was likely confounded by underlying factors associated with both maternal valproate use and the child’s risk of epilepsy (eg, the type of maternal epilepsy). Although due to sample size, our possibilities to perform in-depth analyses were more limited for children with prenatal topiramate and clonazepam exposure, we speculate that similar mechanisms (ie, confounding) may account for the associations with these ASMs. However, the analyses of clonazepam were especially limited by low numbers, and since the mechanism of action (ie, γ-aminobutyric acid receptor activation)^20^ is different from that of topiramate and valproate, we cannot rule out that the association with clonazepam could reflect a true association.

Another interesting finding that relates to the broader question of the teratogenicity of valproate stems from the sibling analyses of malformations. These analyses showed that 6.1% of siblings of children exposed to valproate who were themselves unexposed were diagnosed with major malformations, which is significantly higher than the approximately 3% of children unexposed to ASMs in the general population.^35^ This has, to our knowledge, not been reported before and gives rise to questions regarding the role of genetic, epigenetic, or other environmental factors in risk of adverse offspring outcomes in women with epilepsies previously treated or treatable with valproate. It is also possible that the siblings were not truly unexposed (eg, if the mother had stockpiled medication before pregnancy and therefore did not fill new prescriptions in the exposure window).

In the present study, risk of epilepsy in children of mothers with epilepsy ranged from 2.7% to 9.1% at 15 years of age, which is significantly higher than among children in the general population (<1% at 15 years of age)^29,36^ but resembles the range of estimates reported from other population-based studies of familial epilepsy risk.^29,30,31,37^ These studies have found that risk of epilepsy in children of parents with epilepsy depended on the type of parental epilepsy.^29,30,37^ For instance, in the Rochester Epidemiology Project, the 40-year cumulative risk of epilepsy was reported to be 7.3% for children of parents with generalized epilepsy vs 2.9% in children of parents with focal epilepsy.^30^ Furthermore, while Ottman et al^31^ did not report cumulative risks of epilepsy in children by maternal use of specific ASMs, they did compare any with no use and found no association with offspring seizure risk.^31^ Thus, it is plausible that the variation in epilepsy risk according to maternal ASM use found in our study may reflect variation in genetic risk for specific epilepsy types that are associated with the use of specific ASMs.

### Limitations

This study has limitations. We relied on register-based identification of epilepsy in children and their mothers, and some misclassification of their epilepsy status was possible.^38,39^ In addition, classification of the subtype of maternal epilepsy (a key confounder in this study) was challenging due to the low validity of *ICD-10* codes for epilepsy subclassification,^39^ and sufficient adjustment for the subtype of maternal epilepsy was consequently difficult. For this reason, we performed several sensitivity analyses using approaches that were more robust to confounding by indication, such as the sibling analysis and the use of the discontinuers as the reference. However, these sensitivity analyses were based on smaller groups, which limited the statistical power to detect between-group differences. Another limitation was the use of maternal prescription fills as a proxy for prenatal ASM exposure, and although high levels of adherence to these medications have been reported,^40^ some misclassification was possible. Nevertheless, the quality of the data in the national prescription registers was considered to be high. The reimbursement structure in the Nordic health care systems provided a strong economic incentive for recording all dispensed drugs and ensured high sensitivity in capturing medication exposures.^41^ Furthermore, we estimated mean daily dose by dividing the cumulative amount dispensed by the length of the entire pregnancy. This may have resulted in underestimation of daily dose for mothers who discontinued early in pregnancy, which could have further diluted dose-response associations. We did, however, find similar patterns when considering the cumulative dose, and we validated the sensitivity of our algorithm to detect adverse effects of prenatal valproate exposure by demonstrating dose dependency with known valproate-associated risks (ASD and major malformations), suggesting that we should have been able to detect a dose association with epilepsy as well had there been one. Finally, while it would have been of interest to also study the association in a population without epilepsy (eg, children of mothers using valproate for bipolar disorder or migraine), this was not possible with our data, since the number of children who developed epilepsy in these groups was too low for any meaningful analysis.

## Conclusions

This cohort study found associations between prenatal exposure to valproate and certain other ASMs with epilepsy in children of mothers with epilepsy. However, these associations attenuated upon further analyses of discordant siblings and children of mothers who discontinued ASM treatment prior to pregnancy. These findings suggest that prenatal ASM exposure may not increase epilepsy risk in children of mothers with epilepsy and indicate that differences were more likely associated with other underlying factors (eg, possibly the heritability of the maternal epilepsy).

## Notes

Supplement 1.
eMethods.
**eTable 1.** Characteristics of Children According to Prenatal Antiseizure Medication (ASM) Exposure, Based on Children of Mothers With Epilepsy in 5 Nordic Countries (1996-2017) **eTable 2.** Association of Prenatal Exposure to Valproate and Other Antiseizure Medication (ASM) and Epilepsy, Based on 25 138 Children of Mothers With Active Epilepsy in 5 Nordic Countries (1996-2017) **eTable 3.** Association of Different Cumulative Doses of Prenatal Exposure to Valproate and Other Antiseizure Medication (ASM) and Childhood Epilepsy Based on 38 663 Children of Mothers With Epilepsy in 5 Nordic Countries (1996-2017) **eTable 4.** Sibling Analyses of the Association of Prenatal Valproate Exposure and Autism Spectrum Disorder (ASD) Based on 13 886 Sibling Sets of Mothers With Epilepsy in 5 Nordic Countries (1996-2017) **eTable 5.** Sibling Analyses of the Association of Prenatal Valproate Exposure and Major Malformations Based on 13 886 Sibling Sets of Mothers With Epilepsy in 5 Nordic Countries (1996-2017) **eFigure.** Flowchart of the Study Population

Supplement 2.
Data Sharing Statement
