# Supplementary material for: Prenatal Exposure to Antiseizure Medications and Risk of Epilepsy in Children of Mothers With Epilepsy
Source: JAMA Netw Open. 2024 Feb 26;7(2):e2356425. doi: 10.1001/jamanetworkopen.2023.56425 (PMC10897746; doi:10.1001/jamanetworkopen.2023.56425)
Supplement: Supplement 1. — eMethods. eTable 1. Characteristics of Children According to Prenatal Antiseizure Medication (ASM) Exposure, Based on Children of Mothers With Epilepsy in 5 Nordic Countries (1996-2017) eTable 2. Association of Prenatal Exposure to Valproate and Other Antiseizure Medication (ASM) and Epilepsy, Based on 25 138 Children of Mothers With Active Epilepsy in 5 Nordic Countries (1996-2017) eTable 3. Association of Different Cumulative Doses of Prenatal Exposure to Valproate and Other Antiseizure Medication (ASM) and Childhood Epilepsy Based on 38 663 Children of Mothers With Epilepsy in 5 Nordic Countries (1996-2017) eTable 4. Sibling Analyses of the Association of Prenatal Valproate Exposure and Autism Spectrum Disorder (ASD) Based on 13 886 Sibling Sets of Mothers With Epilepsy in 5 Nordic Countries (1996-2017) eTable 5. Sibling Analyses of the Association of Prenatal Valproate Exposure and Major Malformations Based on 13 886 Sibling Sets of Mothers With Epilepsy in 5 Nordic Countries (1996-2017) eFigure. Flowchart of the Study Population [file jamanetwopen-e2356425-s001.pdf]

Supplemental Online Content

Dreier JW, Christensen J, Igland J, et al. Prenatal exposure to antiseizure medications and risk of epilepsy in children of mothers with epilepsy. *JAMA Netw Open*. 2024;7(2):e2356425. doi:10.1001/jamanetworkopen.2023.56425

eMethods
eTable 1. Characteristics of Children According to Prenatal Antiseizure Medication (ASM) Exposure, Based on Children of Mothers With Epilepsy in 5 Nordic Countries (1996-2017)
eTable 2. Association of Prenatal Exposure to Valproate and Other Antiseizure Medication (ASM) and Epilepsy, Based on 25 138 Children of Mothers With Active Epilepsy in 5 Nordic Countries (1996-2017)
eTable 3. Association of Different Cumulative Doses of Prenatal Exposure to Valproate and Other Antiseizure Medication (ASM) and Childhood Epilepsy Based on 38 663 Children of Mothers With Epilepsy in 5 Nordic Countries (1996-2017)
eTable 4. Sibling Analyses of the Association of Prenatal Valproate Exposure and Autism Spectrum Disorder (ASD) Based on 13 886 Sibling Sets of Mothers With Epilepsy in 5 Nordic Countries (1996-2017)
eTable 5. Sibling Analyses of the Association of Prenatal Valproate Exposure and Major Malformations Based on 13 886 Sibling Sets of Mothers With Epilepsy in 5 Nordic Countries (1996-2017)
eFigure. Flowchart of the Study Population

This supplemental material has been provided by the authors to give readers additional information about their work.

© 2024 Dreier JW et al. *JAMA Network Open*.

eMethods

Calculations of antiseizure medication dose

We calculated the estimated average daily dose of ASM for each monotherapy as the sum of the defined daily doses (DDDs) from all prescriptions filled in the exposure window (30 days before the last menstrual period until birth) divided by the number of days in that period. The DDDs were obtained from the World Health Organization (https://www.whocc.no/atc_ddd_index/; valproate = 1500 mg/d; lamotrigine = 300 mg/d; levetiracetam = 1500 mg/d; carbamazepine = 1000 mg/d; oxcarbazepine = 1000 mg/d; topiramate = 300 mg/d; clonazepam = 8 mg/d) and refer to the assumed average maintenance dose per day for a drug used for its main indication in adults. We categorized the estimated daily ASM dose as high (≥100% DDD), medium (≥50% and <100% DDD), or low (<50% DDD). For example, for valproate, this corresponds to low: <750 mg; medium: 750-1499 mg/d; and high: ≥1500 mg/d. For some of the less commonly used ASMs, it was necessary to combine the medium and high dose groups to ensure sufficient numbers for analysis.

© 2024 Dreier JW et al. *JAMA Network Open*.

eTable 1. Characteristics of Children According to Prenatal Antiseizure Medication (ASM) Exposure, Based on Children of Mothers With Epilepsy in 5 Nordic Countries (1996-2017)

	Prenatal ASM exposure				
	None<br>*n = 22,207*	Lamotrigine monotherapy<br>N = 5,289	Levetiracetam monotherapy<br>N = 1,061	Carbamazepine monotherapy<br>N = 2,664	Oxcarbazepine monotherapy<br>N = 1,460
**Country of birth**					
Denmark	9,385 (42.3)	1,966 (37.2)	285 (26.9)	368 (13.8)	385 (26.4)
Finland	1,225 (5.5)	523 (9.9)	175 (16.5)	1,046 (39.3)	948 (64.9)
Iceland	91 (0.4)	40 (0.8)	14 (1.3)	40 (1.5)	13 (0.9)
Norway	6,101 (27.5)	1,251 (23.7)	301 (28.4)	350 (13.1)	61 (4.2)
Sweden	5,405 (24.3)	1,509 (28.5)	286 (27.0)	860 (32.3)	53 (3.6)
**Year of birth**					
1996-1999	401 (1.8)	61 (1.2)	0 (0.0)	328 (12.3)	146 (10.0)
2000-2004	1457 (6.6)	313 (5.9)	7 (0.7)	492 (18.5)	342 (23.4)
2005-2009	6,482 (29.2)	1,554 (29.4)	155 (14.6)	873 (32.8)	388 (26.6)
2010-2014	8,552 (38.5)	2,083 (39.4)	468 (44.1)	704 (26.4)	417 (28.6)
2015-2017	5,315 (23.9)	1,278 (24.2)	431 (40.6)	267 (10.0)	167 (11.4)
**Sex of child, male**	11,386 (51.3)	2,683 (50.7)	522 (49.2)	1,378 (51.7)	779 (53.4)
**Gestational age at birth <37 weeks**	1,501 (6.8)	359 (6.8)	69 (6.5)	176 (6.6)	81 (5.5)
**Birth weight <2500 g**	1,001 (4.5)	223 (4.2)	47 (4.4)	149 (5.6)	70 (4.8)
**Maternal age, years**					
<20	498 (2.2)	84 (1.6)	16 (1.5)	33 (1.2)	49 (3.4)
20-24	3,608 (16.2)	671 (12.7)	156 (14.7)	248 (9.3)	241 (16.5)
25-29	6,945 (31.3)	1,690 (32.0)	358 (33.7)	771 (28.9)	495 (33.9)
30-34	6,960 (31.3)	1,834 (34.7)	354 (33.4)	945 (35.5)	457 (31.3)
35-39	3,483 (15.7)	853 (16.1)	153 (14.4)	538 (20.2)	175 (12.0)
≥40	713 (3.2)	157 (3.0)	24 (2.3)	129 (4.8)	43 (2.9)
**Maternal parity**					
0	9,834 (44.3)	2,682 (50.7)	552 (52.0)	987 (37.0)	676 (46.3)
1	7,713 (34.7)	1,815 (34.3)	352 (33.2)	956 (35.9)	488 (33.4)
≥2	4,607 (20.7)	785 (14.8)	157 (14.8)	697 (26.2)	280 (19.2)
Missing	53 (0.2)	7 (0.1)	<5[b]	24 (0.9)	16 (1.1)
**Maternal education**					
Compulsory	5,832 (26.3)	972 (18.4)	181 (17.1)	422 (15.8)	261 (17.9)
Secondary/pre-university	9,508 (42.8)	2,434 (46.0)	462 (43.5)	1,376 (51.7)	731 (50.1)
Bachelor	4,230 (19.0)	1,251 (23.7)	271 (25.5)	477 (17.9)	279 (19.1)
Master/PhD	2,057 (9.3)	545 (10.3)	108 (10.2)	276 (10.4)	117 (8.0)

© 2024 Dreier JW et al. *JAMA Network Open*.

Missing	580 (2.6)	87 (1.6)	39 (3.7)	113 (4.2)	72 (4.9)
**Smoking in pregnancy**					
No	16,824 (75.8)	4,230 (80.0)	876 (82.6)	2,175 (81.6)	1,109 (76.0)
Yes	3,931 (17.7)	714 (13.5)	102 (9.6)	276 (10.4)	259 (17.7)
Missing	1,452 (6.5)	345 (6.5)	83 (7.8)	213 (8.0)	92 (6.3)
**Use of antidepressants in pregnancy**	1,631 (7.3)	584 (11.0)	42 (4.0)	118 (4.4)	68 (4.7)
**Maternal psychiatric disorder**[a]	5,974 (26.9)	1,250 (23.6)	180 (17.0)	283 (10.6)	155 (10.6)

[a]Any maternal diagnosis of a psychiatric disorder (ICD-10: F00-F99) registered in the patient registers before birth of the child (DK inpatient contacts since 1994 and outpatient/emergency room contacts since 1995; FI: Inpatient contacts since 1996 and outpatient contacts in public hospitals since 1998; IS: Inpatient contacts since 2002 and outpatient contacts since 2010; NO: Outpatient and inpatient contacts and data from contracted private specialists since 2008; SE: outpatient and inpatient contacts since 2005)

[b] Observations added to the largest category

© 2024 Dreier JW et al. *JAMA Network Open*.

eTable 2. Association of Prenatal Exposure to Valproate and Other Antiseizure Medication (ASM) and Epilepsy, Based on 25 138 Children of Mothers With Active Epilepsy in 5 Nordic Countries (1996-2017)

**Exposure**	***n***	**Number with epilepsy** ***n***	**Incidence rate per 10,000 person-years (95% CI)**	**Basic adjusted[a] HR (95% CI)**	**Fully adjusted[b] HR (95% CI)**	**Cumulative incidence, age 10 %, (95% CI)**	**Cumulative incidence, age 15 %, (95% CI)**
No ASM	9,762	227	31.9 (28.0-36.3)	1.00 (ref)	1.00 (ref)	3.1 (2.7-3.5)	NA
Any ASM[c]	15,376	462	37.7 (34.4-41.3)	1.15 (0.97-1.37)	1.18 (0.99-1.40)	3.7 (3.3-4.0)	5.2 (4.7-5.8)
Monotherapies							
Valproate	1,851	110	60.2 (49.9-72.6)	1.92 (1.46-2.53)	1.96 (1.49-2.59)	5.8 (4.6-7.0)	8.6 (6.9-10.4)
Lamotrigine	4,826	99	31.7 (26.0-38.6)	1.01 (0.79-1.30)	1.03 (0.80-1.32)	3.2 (2.5-3.9)	NA
Levetiracetam	1,043	16	35.6 (21.8-58.2)	1.01 (0.60-1.72)	1.03 (0.61-1.76)	NA	NA
Carbamazepine	2,560	81[d]	30.5 (24.4-38.1)	1.01 (0.75-1.36)	1.07 (0.79-1.45)	2.9 (2.2-3.7)	4.3 (3.3-5.5)
Oxcarbazepine	1,388	27[d]	16.9 (11.3-25.5)	0.62 (0.38-1.02)	0.64 (0.39-1.05)	1.6 (0.9-2.5)	2.4 (1.4-3.7)
Topiramate	260	12[d]	61.8 (34.2-111.7)	1.96 (1.06-3.64)	1.89 (1.01-3.51)	NA	NA
Clonazepam	274	18[d]	48.1 (28.5-81.2)	1.47 (0.83-2.62)	1.48 (0.82-2.65)	4.2 (2.2-7.4)	NA
Polytherapies							
Without valproate	2,012	62[d]	40.6 (31.5-52.3)	1.28 (0.95-1.73)	1.23 (0.91-1.66)	4.6 (3.5-5.9)	NA
With valproate	801	47[d]	62.7 (46.8-84.0)	1.96 (1.37-2.80)	1.93 (1.34-2.77)	5.7 (4.0-7.7)	8.6 (6.0-11.7)

NA = Not analyzed due to low numbers or insufficient follow-up time
[a] Adjusted for year of birth, sex of the child, and country of birth
[b] Additionally adjusted for maternal age, parity, education, smoking in pregnancy, use of antidepressants in pregnancy, and maternal psychiatric comorbidity.
[c] Numbers in mono-and polytherapy do not add up to any ASM exposure, since the following monotherapies are not included due to low numbers: gabapentin, pregabalin, eslicarbazepine, lacosamide, acetazolamide, phenobarbital, and phenytoin.
[d] Numbers copied from Table 2, since the difference in observations between Table 2 and eTable 2 was <5 for this cell and therefore cannot be shown for data privacy reasons. Analyses are however based on actual numbers.

© 2024 Dreier JW et al. *JAMA Network Open*.

eTable 3. Association of Different Cumulative Doses of Prenatal Exposure to Valproate and Other Antiseizure Medication (ASM) and Childhood Epilepsy Based on 38 663 Children of Mothers With Epilepsy in 5 Nordic Countries (1996-2017)

Cumulative exposure[c]	*n*	**Number with epilepsy** *n*	**Incidence rate, per 10,000 person-years** (95% CI)	**Basic adjusted[a] HR** (95% CI)	**Fully adjusted[b] HR** (95% CI)
No ASM	22,207	390	26.5 (24.0-29.2)	1.00 (ref)	1.00 (ref)
**Valproate monotherapy**					
<100 days	489	32	67.3 (47.6-95.1)	2.37 (1.62-3.45)	2.26 (1.55-3.30)
100-199 days	833	45	55.9 (41.7-74.8)	1.95 (1.38-2.74)	1.98 (1.40-2.80)
≥200 days	630	45	68.1 (50.8-91.1)	2.23 (1.57-3.18)	2.34 (1.64-3.33)
**Lamotrigine monotherapy**					
<100 days	1093	22	27.8 (18.3-42.2)	1.08 (0.70-1.66)	1.03 (0.67-1.59)
100-199 days	1272	29	33.4 (23.2-48.1)	1.32 (0.90-1.93)	1.37 (0.93-2.00)
≥200 days	2924	53	30.1 (23.0-39.4)	1.11 (0.83-1.48)	1.17 (0.87-1.57)
**Levetiracetam monotherapy**					
<100 days	155	<5 [d]	NA	NA	NA
100-199 days	205	10 [d]	72.2 (32.4-160.7)	2.74 (1.22-6.18)	2.64 (1.17-5.97)
≥200 days	701	10 [d]	22.7 (10.8-47.5)	0.81 (0.38-1.74)	0.85 (0.40-1.81)
**Carbamazepine monotherapy**					
<100 days	534	11	22.5 (12.5-40.7)	0.78 (0.42-1.44)	0.77 (0.42-1.43)
100-199 days	1089	39	33.7 (24.6-46.1)	1.14 (0.79-1.66)	1.28 (0.88-1.86)
≥200 days	1041	31	30.4 (21.4-43.3)	1.08 (0.73-1.59)	1.19 (0.80-1.76)
**Oxcarbazepine monotherapy**					
<100 days	160	<5 [d]	NA	NA	NA
≥100 days	1300	30 [d]	19.3 (13.1-28.6)	0.68 (0.43-1.08)	0.71 (0.45-1.13)
**Topiramate monotherapy**					
<100 days	157	<5 [d]	NA	NA	NA
≥100 days	133	10 [d]	101.1 (52.6-194.3)	3.52 (1.80-6.88)	3.66 (1.86-7.18)
**Clonazepam monotherapy**					
<100 days	272	12	41.0 (23.3-72.3)	1.61 (0.89-2.89)	1.60 (0.89-2.90)
≥100 days	67	6	82.8 (37.2-184.2)	2.95 (1.29-6.75)	3.10 (1.35-7.13)

NA = Not analyzed due to low numbers or insufficient follow-up time

[a] Adjusted for year of birth, sex of the child, and country of birth

[b] Additionally adjusted for maternal age, parity, education, smoking in pregnancy, use of antidepressants in pregnancy, and maternal psychiatric comorbidity.

[c] The cumulative exposure is estimated as the sum of the defined daily doses (DDDs) from all prescriptions filled in the exposure window (30 days before the last menstrual period until birth) and refer to the number of treatment days with a dose equivalent to the DDD.

[d] Numbers are rounded for data privacy reasons

© 2024 Dreier JW et al. *JAMA Network Open*.

eTable 4. Sibling Analyses of the Association of Prenatal Valproate Exposure and Autism Spectrum Disorder (ASD) Based on 13 886 Sibling Sets of Mothers With Epilepsy in 5 Nordic Countries (1996-2017)

		**Number with ASD**	**Incidence rate, per 10,000 person-years**	**Unadjusted HR**	**Adjusted[a] HR**
Exposure	*n*	*n*	(95% CI)	(95% CI)	(95% CI)
**Sibling sets of mothers using valproate in at least one pregnancy and no valproate in at least one other**	418				
Pregnancies with no valproate use	604	11	19.2 (10.6-34.6)	1.00 (ref)	1.00 (ref)
Pregnancies with valproate use	529	32	55.4 (39.2-78.3)	4.47 (1.62-12.28)	6.41 (2.00-20.58)

[a] Adjusted for sex of the child, maternal age, parity, smoking in pregnancy, and use of antidepressants in pregnancy

eTable 5. Sibling Analyses of the Association of Prenatal Valproate Exposure and Major Malformations Based on 13 886 Sibling Sets of Mothers With Epilepsy in 5 Nordic Countries (1996-2017)

		**Number with major malformations**	**Proportion**	**Unadjusted RR**	**Adjusted[a] RR**
Exposure	*n*	*n*	%	(95% CI)	(95% CI)
**Sibling sets of mothers using valproate in at least one pregnancy and no valproate in at least one other**	418				
Pregnancies with no valproate use	604	37	6.1	1.00 (ref)	1.00 (ref)
Pregnancies with valproate use	529	51	9.6	1.56 (0.97-2.50)	1.66 (1.03-2.67)

[a] Adjusted for sex of the child, maternal age, parity, smoking in pregnancy, and use of antidepressants in pregnancy

© 2024 Dreier JW et al. *JAMA Network Open*.

eFigure. Flowchart of the Study Population

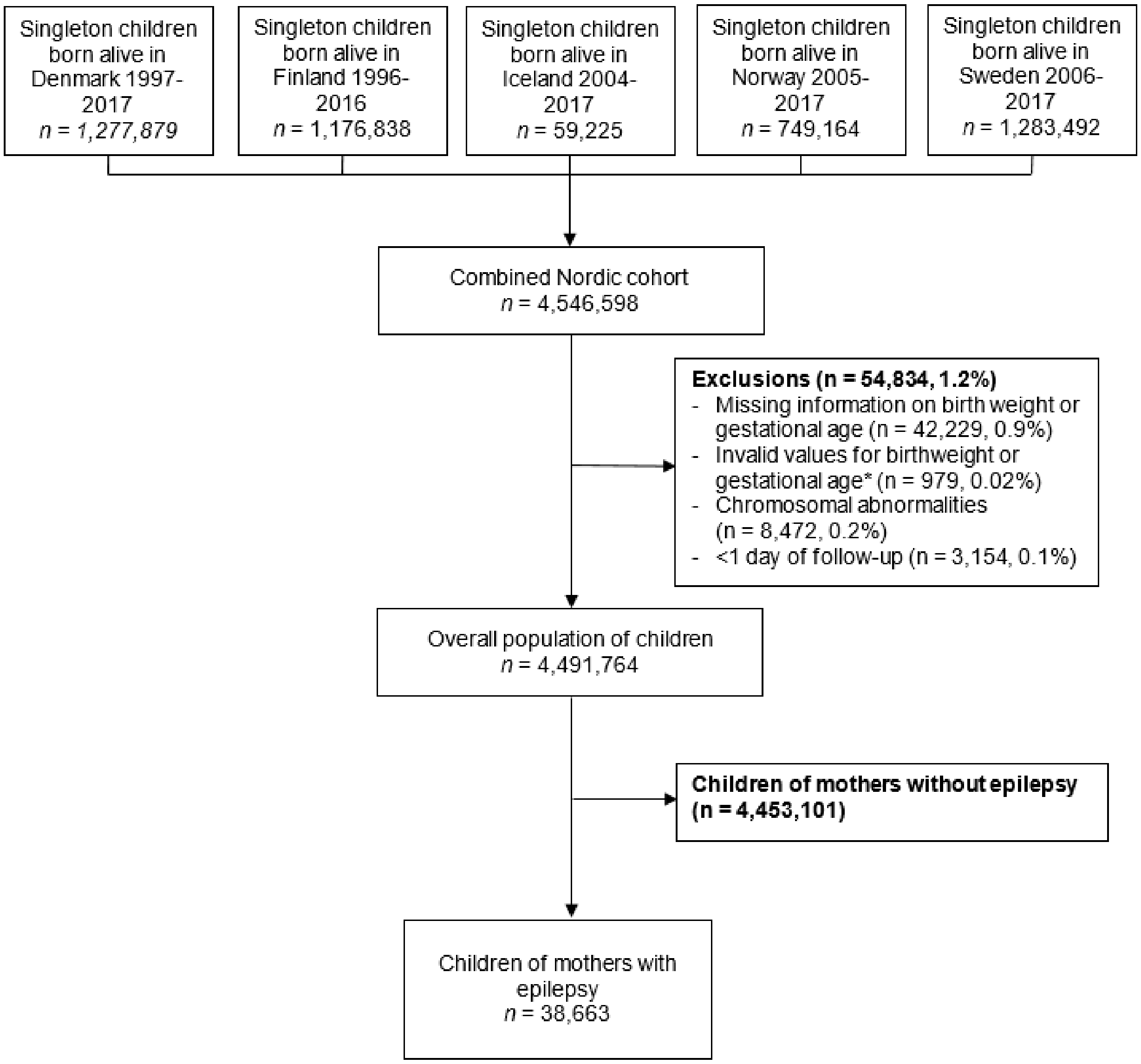

* Invalid values were defined as birth weight <300 gram or >7.000 gram; gestational week <22 or ≥45; or if country- and sex-specific *z*-score for birth weight >4 in children with gestational week <35.

© 2024 Dreier JW et al. *JAMA Network Open*.
