# Supplementary material for: Prenatal Exposure to Antiseizure Medications and Risk of Epilepsy in Children of Mothers With Epilepsy
Source: JAMA Netw Open. 2024 Feb 26;7(2):e2356425. doi: 10.1001/jamanetworkopen.2023.56425 (PMC10897746; doi:10.1001/jamanetworkopen.2023.56425)
Supplement: Supplement 2. — Data Sharing Statement [file jamanetwopen-e2356425-s002.pdf]

Data Sharing Statement

Dreier. Prenatal Exposure to Antiseizure Medications and Risk of Epilepsy in Children of Mothers With Epilepsy. *JAMA Netw Open*. Published February 26, 2024.
doi:10.1001/jamanetworkopen.2023.56425

Data
Data available: No

Additional Information
Explanation for why data not available: Data was based on Nordic national registers and individual level data cannot be shared due to national regulations. However, summary statistics in addition to the results provided in the results section and supplementary material, may be provided upon request. Original data are available upon application to the relevant authorities.
